# Egocentric network characteristics of persons with Type 1 diabetes and their relationships to perceived social support and well-being

**DOI:** 10.1080/21642850.2021.1951272

**Published:** 2021-07-19

**Authors:** Tian Cheyne, Michael A. Smith, Thomas V. Pollet

**Affiliations:** Department of Psychology, Northumbria University, Newcastle upon Tyne, UK

**Keywords:** Egocentric network analysis, well-being, diabetes, multilevel models, social support

## Abstract

**Objectives:** The size of one's support network is positively related to health and well-being. It is therefore important to understand this association in people with Type 1 diabetes, as this could inform interventions. Moreover, the type of support (emotional, instrumental, informational) offered likely varies by gender of both the person seeking support and offering support. We thus examine the relationship between the composition of (perceived) social support networks and well-being in a sample of 121 persons with Type 1 diabetes.

**Design:** An egocentric social network survey, combined with survey measures.

**Main outcome(s):** The size and composition of support networks and well-being.

**Measures:** Participants indicated the type of support individuals in their contact network offered and their gender, alongside measures of perceived social support and well-being. They indicated which individuals offered which types of support (emotional, instrumental, informational).

**Results:** Perceived support was associated with the actual size of the emotional support network. Further, the size of the emotional support network was associated with well-being. Using multilevel models we examined assortment by gender in social support networks. Compared to women, men were more inclined to list the opposite gender as support, especially for emotional and informational support.

**Conclusion:** Mapping out an individual's multidimensional support network paints a more complete picture of support than single item measures of support. We therefore recommend relying on a social network methodology to gain a more complete understanding of support networks. The findings highlight that an association exists between emotional network size and wellbeing. Given the potential implications of this finding for the quality of life of diabetes patients, it is important to establish the causality of this relationship.

Social support has been associated with numerous physical health outcomes (Berkman & Glass, 2000; House, Landis, & Umberson, 1988; Wills & Ainette, 2012), potentially due to its role in buffering against the adverse effects of stress (e.g. Uchino, Cacioppo, & Kiecolt-Glaser, 1996; Uchino, Carlisle, Birmingham, & Vaughn, 2011). In Type 2 diabetes, it has been suggested that social support influences a range of patient outcomes, including self-care (van Dam, van der Horst, Knoops, Ryckman, & Crebolder, 2005), glycaemic control (e.g. Heisler, Vijan, Makki, & Piette, 2010), health behaviours (such as diet and exercise behaviours and frequency of foot examinations, Schiøtz, Bøgelund, Almdal, Jensen, & Willaing, 2012), medication adherence and health decision making (Karlsen, Idsoe, Hanestad, Murberg, & Bru, 2004; Karlsen, Oftedal, & Bru, 2012; Strom & Egede, 2012). With respect to psychological health outcomes, social support has been implicated as a buffer against depression (Littlefield, Rodin, Murray, & Craven, 1990), diabetes-related distress (Baek, Tanenbaum, & Gonzalez, 2014; Schiøtz et al., 2012) and mortality (Ciechanowski et al., 2010). However, it has been argued that due to unique causes of and treatments for Type 1 and Type 2 diabetes, relationships between social support and health behaviours for the these two conditions may be different (Hempler, Joensen, & Willaing, 2016). Despite this, there have been fewer studies which have investigated the role of social support on patient outcomes in Type 1 diabetes, relative to Type 2.

Type 1 diabetes involves a complex treatment regime, incorporating frequent blood glucose monitoring and self-administration of insulin. Although it has been suggested that social support can enhance Type 1 diabetes self-management (Brady, Song, & James Butler, 2017), most studies investigating the role of social support on patient outcomes in Type 1 diabetes have been conducted in adolescent participants. These studies have suggested a role for social support in enhancing well-being and diabetes self-management (Skinner & Hampson, 1998; Skinner, John, & Hampson, 2000). In adults with Type 1 diabetes, living without a partner is associated with poorer health-related quality of life and glycaemic control (Joensen, Almdal, & Willaing, 2013), while attendance at support groups for young adults with Type 1 diabetes is associated with improvements in diabetes self-care and glycaemic control (Markowitz & Laffel, 2012). Cross-sectional self-report data provides further evidence that a relationship exists between social support and emotional well-being in adults with Type 1 diabetes (Joensen, Almdal, & Willaing, 2016).

While these aforementioned studies suggest that social support plays an important role in a range of health and well-being outcomes in people with Type 1 diabetes, a limitation of these studies is that they tend to focus on ‘general social support’, without differentiating between different types of social support. Most conceptualisations of social support recognise that it is multidimensional in nature (e.g. House, 1981). For example, Schaefer, Coyne, and Lazarus (1981) argued for three dimensions of social support: emotional, instrumental and informational support (also see Heaney & Israel, 2008). Emotional support relates to needs in terms of empathy, love, understanding, trust, and caring. Instrumental support involves the provision of aid (e.g. money) and services that directly assist a person in need (e.g. provision of child care or helping to move house). Informational support is the provision of advice and/or information, which a person can use to solve problems. Additionally, some studies in the diabetes literature have measured social support using a single item Visual Analog Scale measure (Griffith, Field, & Lustman, 1990) or a single dichotomous item (whether participants could get help from others when faced with severe illness) (Joensen et al., 2016). Other studies (e.g. Baek et al., 2014; Kaplan & Hartwell, 1987) have used a brief social support questionnaire (Sarason, Sarason, Shearin, & Pierce, 1987) which provides richer detail on the size of an individual's support network and indicates how satisfied they are with the support they receive, but also neglects the nature of the different types of support offered. However, it is critically important to understand the extent to which individuals with Type 1 diabetes feel supported in different contexts, for example when dealing with a hypoglycaemic episode (instrumental support) or for advice on managing their condition (informational support) which is not captured via the social support measures often used in this field. One way of addressing this gap is to employ a social network perspective (reviews in Barrera, 2000; Heaney & Israel, 2008) to ascertain who individuals turn to for support and what type of support these individuals offer. Therefore, our research aims to explore the relationships between a self-report measure of perceived social support, and a social network generator approach whereby a participant (‘Ego’) lists the individuals in their contact network (‘Alters’), and the types of support which these individuals provide. Moreover, we aim to explore how both approaches to measuring social support relate to mental well-being in people with Type 1 diabetes.

These aims will be explored using a dataset collected as part of a student project. This dataset also enables us to explore some questions in relation to the role of gender in the social support networks of individuals with Type 1 diabetes. Previous work on social networks suggests that there are differences between men and women with respect to the size (e.g. Dunbar & Spoors, 1995; McLaughlin, Vagenas, Pachana, Begum, & Dobson, 2010) and quality (e.g. Shumaker & Hill, 1991; Stokes & Levin, 1986) of social networks, as well as the frequency with which social support is sought (e.g. Belle, 1987; Day & Livingstone, 2003; Shumaker & Hill, 1991). In the area of diabetes, there is research focussing on the role of gender in *specific* social relationships, such as between spouses (e.g. August, Rook, Franks, & Parris Stephens, 2013), but data on larger support networks is missing. Therefore, this study aims to explore the role of gender for the entire support network. In sum, we were interested in exploring whether:

(1) men list fewer people they can turn to for support than women;
(2) listed social support networks are associated with self-report measures of (i) perceived social support and (ii) mental well-being;
(3) people with Type 1 diabetes turn to people of the same gender for support or whether they are more likely to seek support from those of the opposite gender;
(4) women more likely to be named as offering support than men (and whether this is particularly the case for emotional support);
(5) there is a relationship between gender and multiplexity (i.e. being listed as more than type of support network member) with women more likely to be listed as offering support.

Due to the exploratory nature of our research we did not establish any hypotheses. However, the student project for which this dataset was collected did pre-register one hypothesis, so for completeness and transparency we will report the findings in relation to this hypothesis. It was hypothesised that perceived social support would mediate the relationship between gender and mental well-being (6). This hypothesis was predicated by the notion that women might benefit from higher perceived social support which could potentially account for their higher scores on mental well-being, as compared to men (e.g. House et al., 1988).

## 1. Methods

### 1.1. Participants

A total of 243 individuals started a survey on Qualtrics between January 2020 and March 2020. The final sample for analysis comprised 121 participants who completed the survey, the majority were obtained via a link on Reddit (*N*=100). Others were obtained via Facebook (*N*=12), a Diabetes forum (*N*=5), word of mouth (*N*=3) or email (*N*=1). There were 87 women and 34 men (*M*=29.33 years, SD=10.32 years, range: 18 to 62 years). Participants had to confirm that they were over 18 years and had a diagnosis of Type 1 diabetes before they could take part.

### 1.2. Procedure

After providing informed consent, participants (‘Egos’) completed an online survey. Participants first provided some basic sociodemographic data (age and gender). They then completed a network generator where they could list up to 25 network members (‘alters’) and they indicated who provided what type of support. Next, participants provided the gender of each of the listed alters. Participants had the option not to assign gender to these alters. Following these attributions of gender, participants indicated who in their network was also a person with Type 1 diabetes. Finally they completed a measure of perceived social support and a measure on mental-wellbeing, after which they were thanked and debriefed. The procedure was approved by the local ethics committee where the survey was carried out.

### 1.3. Materials

#### 1.3.1. Network generator

Participants were instructed to list up to 25 people who they have been in contact with over the past month (*Please state up to 25 people with whom you have been in contact in the past month. This contact can consist of personal contact, but also contact via telephone, internet or e-mail. The list you provide should include people who you know (think of friends, family, acquaintances, etc.)*). This is similar to commonly used network generators in Perry, Pescosolido, and Borgatti (2018, 83-ff). Research on data collection of social networks via the internet has found that most people tend to list between one and ten people in response to four different network-generating questions, even if allowed to provide up to 30 names (Manfreda, Vehovar, & Hlebec, 2004). Allowing participants to enter up to 25 individuals should therefore be sufficient for most participants to capture their entire network, without overburdening them. We refer to this list of people provided by the participant as the ‘contact network’ and to the people listed as ‘alters’. Our strategy was to gather a larger network of contacts and then identify who people turn to in times of need (similar to Binder, Roberts, & Sutcliffe, 2012; Molho, Roberts, de Vries, & Pollet, 2016). In total 1974 alters were listed; 44 individuals listed 25 people in their contact network (36.36%). On average participants listed 16 alters (Appendix Table A1).

#### 1.3.2. Alter attributes

Participants indicated which individuals from their list provided emotional support, instrumental support and informational support. We followed the definitions by Schaefer et al. (1981). These questions were phrased as follows: *Emotional support is described as the expression of empathy, trust, intimacy and reassurance from another person. From the list that you provided, who would you consider you can confide in during times of need/feeling low?*, *Instrumental support is concerned with the direct aid and support from someone you know. For example, this can include material goods, financial aid, emergency medical supplies and provisional services such as chores, childcare and ordering medical supplies. From the list you provided, who would you consider you can go to for help?* and *Informational support involves advice, suggestions, guidance and/or useful information given by another person (e.g. advice from a doctor on insulin dosages or suggestions from friends on lifestyle choices). From the list of people you provided, who would you consider provides information or advice with your diabetes management?*

Next, participants indicated who in their network was male and next indicated who in their network was female. Note that they had the option not to identify gender of the alter or to indicate that alters were both male and female (8 alters, excluded for alter level analyses, $N_{\mathrm{male}}=802$, $N_{\mathrm{female}}=1054$). 89 out of 121 participants indicated a gender for each of the listed alters.

Finally, they also indicated who in their network also had Type 1 diabetes (*N*_Diabetic_ = 51). 93 out of 121 participants did not list any alters with Type 1 diabetes. Due to time constraints we did not ask participants to list other attributes of alters, such as whether they were kin or not (e.g. Roberts, Dunbar, Pollet, & Kuppens, 2009), or to indicate alter-alter ties, i.e. does this alter know this other alter.

#### 1.3.3. Multidimensional scale of perceived social support (MSPSS)

The MSPSS consists of twelve items (Zimet, Dahlem, Zimet, & Farley, 1988). It is a widely used scale to assess perceived social support and has demonstrated excellent test-retest reliability (e.g. Dahlem, Zimet, & Walker, 1991; Kazarian & McCabe, 1991). Sample items include: *‘I can talk about my problems with my friends’*, *‘my family really tries to help me’* and *‘there is a special person who is around when I am in need’*. All items were answered on a 7-point Likert scale (1 = Very Strongly Disagree to 7 = Very Strongly Agree). The scale showed excellent reliability based on Cronbach's *α* (α=.94). The MSPSS consists of three subscales with four items each (family, friends, significant other), which similarly demonstrated excellent reliability (respective *α*'s: .9, .9, .93). Our focus is on the overall scale but we also present results on the sub-scales.

#### 1.3.4. Warwick-Edinburgh mental well-Being scale (WEMWBS)

The WEMWBS consists of fourteen statements relating to mental health (Tennant et al., 2007). It has been validated across a wide range of populations (e.g. Bass, Dawkin, Muncer, Vigurs, & Bostock, 2016). Sample items are: *‘I've been feeling optimistic about the future’* and *‘I've been feeling close to other people’*. Participants rated each statement describing their experience within the last 2 weeks using a 5-point Likert scale (1 = none of the time to 5 = all of the time). The reliability as assessed via Cronbach's *α* was excellent (α=.93).

#### 1.4. Data analysis

All the analyses were conducted in R 4.0.2 (R Development Core Team, 2008). The data, code (incl. packages used), and analysis document are available from the Open Science Framework (OSF). The design for this project was pre-registered and all analyses, including additional checks and analyses, are fully reported on the OSF. We divided our results section into exploratory and confirmatory analyses. After presenting descriptive statistics, correlations and Welch *t*-tests (Ruxton, 2006), we use OLS regression and mediation models (Revelle, 2016) to examine associations at ego level (Level 2) between ego's characteristics, network size, MSPSS, WEMWBS (for additional analyses see OSF). Testing for gender homophily requires a multilevel model, as we have alters (Level 1) nested in egos (Level 2). We performed multilevel logit models (‘lme4’, Bates, Mächler, Bolker, & Walker, 2014) to examine the interaction between alter's gender (Level 1) and ego's gender (Level 2) on being a member of the emotional, instrumental, and informational support network. A positive coefficient of the interaction would be suggestive of gender homophily. Multiplexity, i.e. being listed as a tie more than once, was assessed with multilevel Poisson models to account for the count distribution (1,2,3). These multilevel logit and Poisson models had a random slope for the gender of the alter (Level 1) nested in Ego's ID variable (Level 2). We used multilevel Bayesian logit and Poisson models to generate the estimates used in the plots (Buerkner, 2015). These were based on four chains, with 4000 iterations each (for more details see OSF page). We use age of the participant (Ego) as a control variable throughout the analyses.

### 1.5. Ethics statement

This research was approved by the local ethics commission at the institution where it was carried out (Ref: 20348).

## 2. Results

### 2.1. Descriptive statistics, *t*-tests, and Pearson correlation matrices

Appendix Table A1 shows the descriptive statistics for Ego. People listed on average 16 alters (of which on average 7 were men and 9 were women).

Participants listed more female than male alters in their contact network, t(120)=3.871, p=.0002, Cohen's *d*=0.352). Out of the 1974 alters listed in the contact network, more than half (*N*=1123) did not fulfil any supporting role, 431 offered one role of support, 280 offered two forms of support and 140 offered all three forms of support. Participants listed on average 5 alters in their emotional support network, around 4.5 alters in their instrumental networks and around 2 alters in their informational network. A paired samples *t*-test showed that emotional support network sizes were significantly larger than informational support networks (t(120)=7.632, *p*=<.0001, Cohen's *d*=0.693). Instrumental support networks were also significantly larger than informational support networks (t(120)=7.468, *p*=<.0001, Cohen's *d*=0.679). Emotional support network sizes were, however, not significantly larger than instrumental support networks (t(120)=1.7, *p*=.091).

Appendix Table A2 summarises the correlations between Ego characteristics (after corrections for multiple testing via Holm's (1979) procedure).

The MSPSS did not significantly relate to the size of the contact network listed. Nor did any of its subscales (all *r*<.13, all *p*'s > .16). MSPSS did relate to the number of alters listed in the emotional support network (r(119)=.336, *p*=.0002) and instrumental support network (r(119)=.304, *p*=.0006), and to a lesser extent the informational support network (r(119)=.185, *p*=.042).

***1: Exploratory analyses: Gender differences in (perceived support) networks and well-being***

Gender was not related to the size of the contact network, emotional support network size, informational support network size and instrumental support network size (all *t*-tests < .75, all *p*'s > .45). Gender was also not significantly related to MSPSS scores (t(51.2)=1.33, *p*=.191). Similarly, gender was not significantly related to any of the sub-scales of the MSPSS (all *t*-tests < 1.4, all *p*'s > .17). Men and women also did not significantly differ in their reported well-being (t(58.42)=1.03, *p*=.31).

***2: Exploratory OLS regression analyses: Support networks and multidimensional perceived social support***

Table 1 shows hierarchical OLS regression models on perceived social support. Model 1 showed a statistically significant effect of the size of the emotional support network on MSPSS, but not for the instrumental and informational support networks. This effect of emotional support network size is upheld after accounting for Gender (*p*=.704) and Age (Table 1; Models 2 and 3), though the effect of age was close to statistical significance (*p*=.063). The effect of the size of the emotional support network is also upheld after bootstrapping with 10,000 bootstraps (Davison & Hinkley, 1997) (Model 1; Bias-corrected and accelerated (Bca) 95% CI for *β*: 0.024 to 0.388). In contrast, the effect of age was not (Model 3; Bias-corrected and accelerated (Bca) 95% CI for *β*: −0.362 to 0.012).

**Table 1.** Hierarchical OLS regression models to predict MSPSS. Standardised coefficients (±SE).

	MSPSS		
	Model 1	Model 2	Model 3
Emotional Support *N*	0.231${}^{*}$ (0.107)	0.230${}^{*}$ (0.107)	0.223${}^{*}$ (0.106)
Instrumental Support *N*	0.140 (0.108)	0.141 (0.108)	0.147 (0.107)
Informational Support *N*	0.111 (0.088)	0.111 (0.088)	0.126 (0.087)
Gender		0.018 (0.048)	
Age			−0.159 (0.085)
*N*	121	121	121
$R^{2}$	0.141	0.142	0.166
Adjusted $R^{2}$	0.119	0.112	0.137
Residual Std. Error	0.935 (df = 118)	0.938 (df = 117)	0.925 (df = 117)
*F* Statistic	6.444${}^{***}$ (df = 3; 118)	4.834${}^{**}$ (df = 4; 117)	5.816${}^{***}$ (df = 4; 117)

${}^{*}p<.05$; ${}^{**}p<.01$; ${}^{***}p<.001$. Note: Model 1 contains three types of support. Model 2 controls for Gender. Model 3 controls for Age.

**Figure 1.**
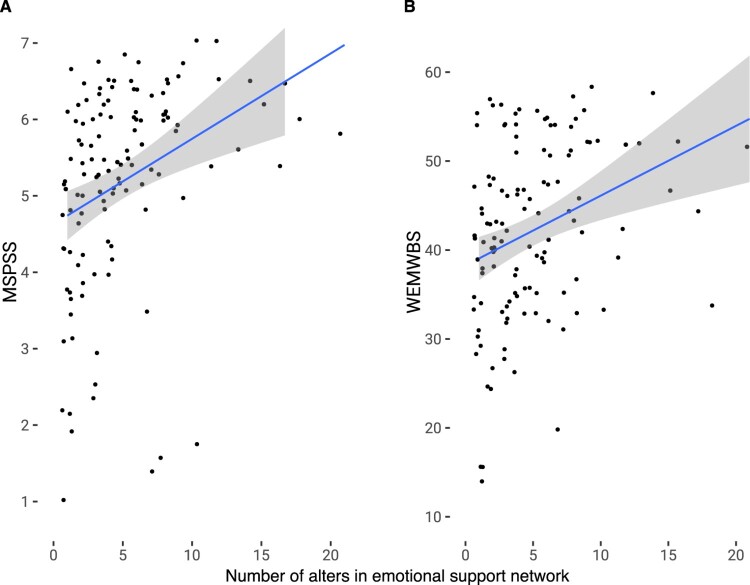
Scatter plots with size of emotional support network and multidimensional perceived social support (MSPSS) and well-being (WEMWBS). Lines are OLS regressions fit with 95% confidence intervals.

Figure 1(A) demonstrates the positive relationship between emotional support network size and multidimensional perceived support from Table 1 (Model 1).

We performed similar analyses to Table 1 for the sub-scales of the MSPSS. The sub-scale analyses are reported in full on the OSF. Emotional support network size predicted MSPSS_family_ (Model 1; Bias-corrected and accelerated 95% CI for *β*: 0.110 to 0.477). Informational support network size predicted MSPSS_friend_ (Model 1; Bca 95% CI for *β*: 0.0601 to 0.2972). None of the support network variables significantly predicted the MSPSS_significant other_ sub-scale (Model 1: all *p*'s > .1).

***2: Exploratory OLS regression analyses: (Perceived) Support networks and well-being***

Table 2 contains hierarchical OLS regression models on well-being. Model 1 demonstrated a significant effect of the size of the emotional support network, but not the size of the instrumental and informational support network (both *p*>.19), on well-being. Model 2 showed the effect of emotional support network was upheld after accounting for the MSPSS, which was also significantly and positively related to well-being. The effects found in Model 2 were upheld after accounting for gender and age (both *p*>.2) in Models 3 and 4. The effects of the emotional support network and MSPSS are also upheld after bootstrapping with 10,000 bootstraps (Model 2; Bca 95% CI for *β*: .021 to .395 and Bca 95% CI for *β*: .096 to .544, respectively).

**Table 2.** Hierarchical OLS regression models to predict MSPSS. Standardised coefficients (±SE).

	WEMWBS			
	Model 1	Model 2	Model 3	Model 4
Emotional Supp. *N*	0.228${}^{*}$ (0.108)	0.205${}^{*}$ (0.087)	0.205${}^{*}$ (0.088)	0.201${}^{*}$ (0.088)
Instrumental Supp. *N*	0.154 (0.110)			
Informational Supp. *N*	−0.036 (0.089)			
MSPSS		0.324${}^{***}$ (0.087)	0.326${}^{***}$ (0.088)	0.338${}^{***}$ (0.088)
Gender			−0.022 (0.046)	
Age				0.088 (0.083)
*N*	121	121	121	121
$R^{2}$	0.113	0.192	0.193	0.199
Adjusted $R^{2}$	0.091	0.178	0.173	0.179
Residual Std. Error	0.950 (df = 118)	0.903 (df = 119)	0.906 (df = 118)	0.902 (df = 118)
*F* Statistic	5.029${}^{**}$ (df = 3; 118)	14.100${}^{***}$ (df = 2; 119)	9.411${}^{***}$ (df = 3; 118)	9.778${}^{***}$ (df = 3; 118)

${}^{*}p<.05$; ${}^{**}p<.01$; ${}^{***}p<.001$. Note: Model 1 contains three types of support. Model 2 retains the emotional support network and adds MSPSS. Model 3 controls for Gender. Model 4 controls for Age.

Figure 1(B) demonstrates the positive relationship between emotional support network size and well-being from Table 2 (Model 2).

We also examined Model 2 from Table 2 with the sub-scales of the MSPSS, rather than the overall score MSPSS. These sub-scale analyses are reported in full on the OSF. All three sub-scales were significantly related to well-being (MSPSS_family_: Bca 95% CI for *β*: 0.012 to 0.364; MSPSS_friend_ Bca 95% CI for *β*: 0.023 to 0.459; MSPSS_significant other_ Bca 95% CI for *β*: 0.022 to 0.429).

### 3-5: Exploratory multilevel logit and Poisson models for support networks

These sets of analyses address whether gender of the ego and the alter influence who is listed as a support network member.

**Figure 2.**
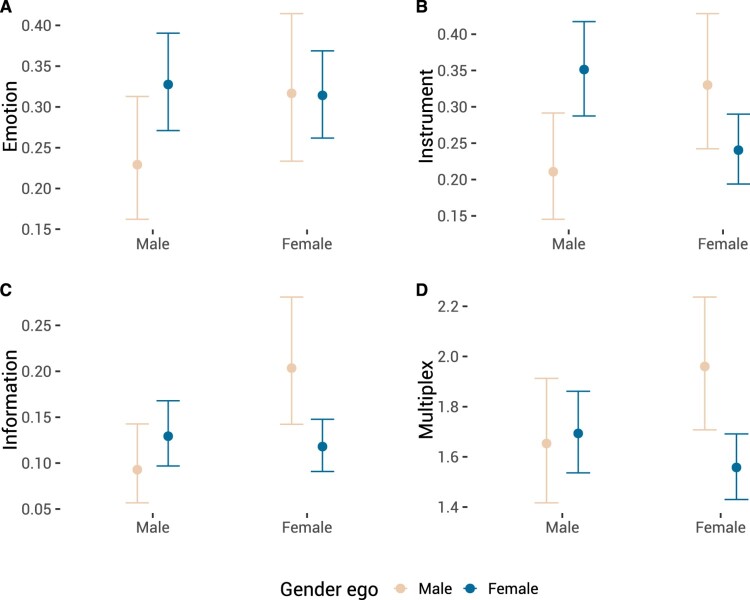
Interaction effects from Table 3 (A: Model 3; B: Model 7; C: Model 11) and Table 4 (D: Model 3). *X*-axis is gender of alter. 95% confidence intervals based on estimates from Bayesian models. Note that the scales of the *Y* -axes vary.

Multilevel logit models for emotional support showed an interaction effect for gender of alter and gender of ego (Table 3: Model 3; Figure 2(A)). This interaction was upheld after running 10,000 simulations following Gelman and Hill's (2006) simulation approach for fixed effects (Median Odds Ratio: 1.662, 95% CI: 1.026 to 2.692). As shown in Figure 2(A), compared to women, men tended to nominate relatively more women as part of their support group.

**Table 3.** Hierarchical multilevel logit models (fixed effects) for support. Coefficients are logits.

	Emotional support				Instrumental support				Informational support			
	Model 1	Model 2	Model 3	Model 4	Model 5	Model 6	Model 7	Model 8	Model 9	Model 10	Model 11	Model 12
Gender Alter	0.09	0.08	0.95${}^{*}$	0.95${}^{*}$	−0.20	−0.22	1.74${}^{***}$	1.73${}^{***}$	0.19	0.23${}^{***}$	1.91${}^{**}$	1.86${}^{***}$
Gender Ego		0.21	0.99${}^{*}$	0.99${}^{*}$		0.23	1.83${}^{***}$	1.84${}^{***}$		−0.25${}^{***}$	1.35${}^{*}$	1.34${}^{***}$
Age				−0.001				0.005				0.01${}^{***}$
Gender Alter * Gender Ego			−0.50${}^{*}$	−0.51${}^{*}$			−1.14${}^{***}$	−1.14${}^{***}$			−0.99${}^{**}$	−0.97${}^{***}$
Constant	−0.97${}^{***}$	−1.30${}^{**}$	−2.65${}^{***}$	−2.65${}^{***}$	−0.63${}^{**}$	−1.01${}^{*}$	−3.74${}^{***}$	−3.75${}^{***}$	−2.21${}^{***}$	−1.84${}^{***}$	−4.55${}^{***}$	−4.50${}^{***}$
*N*	1856	1856	1856	1856	1856	1856	1856	1856	1856	1856	1856	1856
Log Likelihood	−1109.30	−1108.83	−1106.84	−1106.83	−1054.35	−1053.86	−1044.48	−1044.35	−731.15	−730.50	−725.56	−724.84
AIC	2228.61	2229.67	2227.67	2229.66	2118.70	2119.73	2102.96	2104.71	1472.30	1473.01	1465.11	1465.67
BIC	2256.24	2262.82	2266.35	2273.87	2146.33	2152.88	2141.64	2148.92	1499.93	1506.17	1503.80	1509.88

${}^{*}p<.05$; ${}^{**}p<.01$; ${}^{***}p<.001$. Note: Models 1–4 are for Emotional support. Models 5–8 are for Instrumental support. Models 9–12 are for Instrumental support. Each set of models is hierarchical. The first model includes gender of alter (Model 1,5,9), followed by gender of alter (Model 2,6,10), followed by their interaction (Model 3,7,11), followed by Ego's Age (Model 4,8,12).

**Table 4.** Hierarchical multilevel Poisson models (fixed effects).

	Number of types of support offered			
	Model 1	Model 2	Model 3	Model 4
Gender Alter	−0.019	−0.012	0.417	0.429${}^{*}$
Gender Ego		−0.117	0.270	0.292
Age				0.005${}^{*}$
Gender Alter * Gender Ego			−0.250${}^{*}$	−0.258${}^{*}$
Constant	0.541${}^{***}$	0.733${}^{***}$	0.069	0.033
*N*	824	824	824	824
Log Likelihood	−1108.399	−1106.568	−1104.463	−1102.517
AIC	2226.798	2225.137	2222.927	2221.033
BIC	2250.369	2253.422	2255.926	2258.747

${}^{*}p<.05$; ${}^{**}p<.01$; ${}^{***}p<.001$. Note: The first model includes gender of alter (Model 1), followed by gender of alter (Model 2), followed by their interaction (Model 3), followed by Ego's Age (Model 4).

Similar to the findings for emotional support, we also found an interaction effect for instrumental support (Table 3: Model 3; Figure 2(B)). This interaction effect was stronger for men, as men tended to nominate women, whereas women tended to nominate men as providing instrumental support (Based on 10,000 simulations: Median Odds Ratio: 3.13, 95% CI: 1.92 to 5.11).

Table 3 contains multilevel logit models for informational support (Models 9 to 12). However, it should be noted that there were some convergence issues while fitting the models in Table 3 (please see OSF), we therefore paid particular attention to robustness checks. Model 10 from Table 3 suggested that men were less likely to nominate alters than women, whereas women were more likely to be nominated as informational support than men. However, Model 11 demonstrated that this effect is qualified by an interaction effect (Figure 2(C)). This interaction was upheld after running 10,000 simulations (Median Odds Ratio: 2.7, 95% CI: 1.67 to 4.37). Model 12 suggested an age effect with older participants being more inclined to nominate alters. This age effect was not upheld after running 10,000 simulations (Median Odds Ratio: 1.01, 95% CI: 0.99 to 1.03). A Bayesian multilevel logit model supported the interaction effect of gender (Model 11; 95% CI: −1.70 to −0.39), but did not support the age effect (Model 12; 95% CI: −0.01 to 0.03).

Table 4 contains multilevel Poisson models for multiplexity of those alters who are nominated at least once (*N*=824). Model 3 showed a similar interaction pattern as for individual types of support network. Figure 2(D) demonstrates this interaction effect for multiplex ties. Compared to women, who if anything were more inclined to nominate women more so than men, men demonstrated heterophily: men tended to nominate women more often than men. Model 4 also suggested an age effect with older participants assigning more ties to be multiplex. However, further Bayesian multilevel Poisson analyses suggested that this age effect is not robust (Model 4; 95% CI: −0.01 to 0.01; please see OSF).

***6: Confirmatory analysis: Does perceived social support mediate the relationship between gender and mental well-being?***

In our preregistration, we had hypothesised that the MSPSS would mediate the relationship between Gender and the WEMWBS. A mediation model with 5000 bootstraps did not support the existence of this indirect path (bootstrapped 95% CI for standardised ‘ab’ path: −0.03 to 0.14). Therefore, there is no support for a mediation effect.

## 3. Discussion

The present study sought to explore, in individuals with Type 1 diabetes, whether (i) there is concordance between perceived social support and objective social networks, (ii) there are associations between support network sizes and mental well-being and (iii) there are gender differences in terms of social network size, the gender of those sought out to provide support, and the type of support offered (i.e. emotional, instrumental or informational support). Participants tended to list more women than men in their contact network, and reported larger emotional and instrumental support networks than informational support networks. Perceived social support, as measured by the MSPSS, was significantly associated with the size of the individual's emotional support network, but not their instrumental or informational support networks. Similarly, well-being, as measured by the WEMWBS, was significantly related to emotional support network size, but not with the size of the other two types of support network. Interestingly, both emotional and informational support networks were more heterophilous for men than women, indicating that men were more likely to turn to women for emotional and informational support than women were to seek emotional or informational support from men. With respect to instrumental support, the interaction between gender of ego and gender of alter were suggestive of heterophily for both genders. Men are more likely to seek instrumental support from women than men, and women are more likely to seek this type of support from men than women. The pre-registered hypothesis that perceived social support would mediate the relationship between gender and mental well-being was not supported.

In the present study, we addressed gaps in the literature, by using a multidimensional approach to understanding social support in individuals with Type 1 diabetes. What is clear from our findings is that it is critically important to differentiate between emotional, informational and instrumental support in evaluating the influence of social support on well-being. Our finding that perceived social support was related only to the size of an individual's emotional support network indicates that the MSPSS is a useful indicator of the emotional support received by individuals with Type 1 diabetes, but that it may be less adequate for capturing the instrumental and informational support that an individual receives. While a straightforward self-report measure of emotional support is of course highly useful, there may be contexts where it is important to capture gaps in instrumental and informational support in people with Type 1 diabetes. For example, it might be important for people with Type 1 diabetes to have a trusted and reliable network of people to support them if they experience hypoglycaemia and need to be supported to access medical care or supplies (instrumental support) or for advice on how to manage their condition (informational support). However, our findings indicate that such support would not necessarily be captured by self-report instruments such as the MSPSS. Therefore, taking the approach of asking individuals to list the members of their contact network and to indicate the type of support that each individual provides appears to be a more optimal method for capturing multiple dimensions of social support than using only self-report measures such as the MSPSS.

Analysis of the subscale scores on the MSPSS suggested that emotional network sizes were associated with perceived familial support. This further validates our approach, as it would be expected that those with robust emotional support networks would perceive greater support from their family. It might also have been expected that emotional network size would be associated with perceived support from friends. However, our data suggest that perceived support from friends was associated only with informational network sizes. Perhaps this finding is unsurprising, given that it has been suggested that social support from friends can influence health in young people with diabetes, including via the provision of information and resources (Palladino & Helgeson, 2012). However, this interpretation is speculative and further work is warranted to investigate the provision of informational support from friends. Informational support network sizes were significantly smaller than emotional and instrumental support networks, which again makes sense, owing to the fact that participants reported, on average, that less than 0.5 people in their contact network were also diagnosed with diabetes. Therefore, there are likely to be fewer trusted and understanding alters to whom people with Type 1 diabetes feel they can turn to for informational support about understanding and managing their condition.

In concordance with a wealth of existing research suggesting a relationship between perceived social support, health and well-being (Brady et al., 2017; Reblin & Uchino, 2008; Uchino et al., 2011), all three perceived support subscales were significantly related to subjective well-being. This is indicative that perceived support from friends, family members and significant others are all related to well-being in people with Type 1 diabetes (e.g. McPherson, Smith-Lovin, & Cook, 2001). Further, a novel finding to emerge from our exploratory analyses was that despite the notion that ‘likes attract likes’ in social networks, in the present study, men were more likely to seek all three types of support from women than they were from men. This fits with the notion that women are more likely than men to offer social support (Belle, 1987); therefore men may deliberately seek out women to provide social support and women may be more open about providing social support than men. However, with respect to instrumental support women listed proportionately more men as part of their support network. Speculatively, this could be because instrumental support is most likely to be offered by spouses/romantic partners or family members, whereby gender has less of an influence on the likelihood of who is providing support. It is known that spouses are typically involved in their partners' diabetes management, and on this basis are involved in the provision of instrumental support (August et al., 2013). Taken together, our findings provide provisional evidence that gender heterophily is a feature of the social support networks of men with Type 1 diabetes in particular.

### 3.1. Strengths and limitations

A key strength of the present study was that we used a network generator approach to investigate the nature of contact and social networks, which is a novel methodological approach in studies investigating social support in Type 1 diabetes. This allowed us to address a gap in the literature, moving beyond often single item measures of general social support, to a more robust indicator of multidimensional support. We also focussed solely on individuals with Type 1 diabetes, given the focus predominantly in the existing literature on Type 2 diabetes, or a combined group of both Type 1 and Type 2 diabetes patients. This is especially important because of known differences in the ways that social support impacts health outcomes between people with Type 1 and Type 2 diabetes (Hempler et al., 2016). However, the present study is not without its limitations. Our study was cross-sectional and given that we relied on data from a single point in time, it is unclear whether we accurately captured the social network of our participants (e.g. Kogovšek & Ferligoj, 2005). Future research could aim to model changes in the social network and how they relate to changes in well-being, which will allow for a better inference of the potential causal relationships between social support and mental well-being. Moreover, it would be worthwhile to capture other characteristics of the alters, though this adds to the burden of completing the social network survey. In particular, the type of relationship between ego and the alter would be relevant (e.g. neighbour versus friend), but also the closer study of sociodemographic characteristics, such as ethnicity or educational attainment, is necessary. Our sample was obtained online, and it is unclear to what extent our findings would generalise to the population at large. In particular, due to the nature of our data collection we might not have sampled representatively elderly people with Type 1 diabetes or those with more severe symptoms. In future research, online data could be supplemented with targeted sampling strategies to ensure that people with certain sociodemographics are not missed. Finally, an additional limitation is that we relied on a general measure of mental well-being, which allows comparison with other studies which have investigated relationships between social support and well-being. However, future research would benefit from also including a measure related *specifically* to the well-being associated with diabetes management. While we expect a good correspondence between general mental well-being and a diabetes-specific measure, it is possible that the relationship with social support differs for ‘general’ well-being, relative to ‘specific’ management of a chronic illness.

## 4. Conclusion

While our pre-registered hypothesis that perceived social support would mediate the relationship between gender and mental well-being was not supported, our exploratory analyses shed a novel and interesting light on the nature of support networks in people with Type 1 diabetes and their associations with subjective well-being. We found that perceived social support was related to the size of emotional support networks, but not instrumental or informational support networks. Should this finding be replicated, there are important implications for how social support is measured and conceptualised in Type 1 diabetes research and clinical practice. This finding indicates that perceived social support measures which do not capture multidimensional social support, such as the MSPSS, cannot provide a complete picture of the various ways in which an individual is supported by their contact network. Neglecting to measure other dimensions of social support, including instrumental and informational support is problematic, given the importance of these components to diabetes management and coping with the illness. Further, we found that emotional support network size may be an important predictor of subjective well-being in Type 1 diabetes patients, although further work is needed to confirm the causality of this relationship.

Finally, we observed some interesting findings with respect to the gender structure of social networks in individuals with Type 1 diabetes. Men tended to seek all three types of social support more from women than from men. For emotional and informational support, men's networks were more heterophilous than women's. For instrumental support networks, both genders preferred to seek support from the opposite gender than their own gender. Taken together, these findings may tentatively support the idea that (i) women are more likely than men to provide social support, and (ii) spouses are a likely source of instrumental support. While there are implications here in terms of informing research and clinical practice with regard to the way that multidimensional social support networks are structured in Type 1 diabetes, and their role in enhancing well-being, further research is warranted owing to the cross-sectional design and exploratory nature of the analyses.

## Appendix

**Table A1.** Descriptive statistics of Ego networks (*N*=121).

Variable	Mean	SD
Age (years)	29.331	10.298
Contact network	16.314	7.538
Male contacts	6.694	4.179
Female contacts	8.777	5.229
Diabetic contacts	0.421	1.174
Emotional support *N*	5.000	3.964
Instrumental support *N*	4.488	3.302
Informational support network	2.174	1.909
MSPSS: multidimensional scale of perceived social support	5.188	1.319
MSPSS- significant other	5.729	1.608
MSPSS- family	4.878	1.541
MSPSS- friend	4.957	1.370
WEMWBS: Warwick-Edinburgh mental well-being scale	42.190	9.927

**Table A2.** Correlation matrix between Egocentric variables (*N*=121, corrected for multiple testing using Holm's (1979) procedure).

Variable	Age	Altrs	Male *N*	Female *N*	Diabetic *N*	Emot *N*	Ins *N*	Inf *N*	MSPSS	WEMWBS
Age	1.00									
Alters	−0.06	1.00								
Male support *N*	−0.09	0.68${}^{***}$	1.00							
Female support *N*	−0.02	0.76${}^{***}$	0.22	1.00						
Diabetic support *N*	0.04	0.03	0.06	−0.01	1.00					
Emotional support *N*	−0.01	0.49${}^{***}$	0.37${}^{***}$	0.43${}^{***}$	0.02	1.00				
Instrumental support *N*	0.03	0.35${}^{**}$	0.20	0.32${}^{*}$	−0.09	0.60${}^{***}$	1.00			
Informational support *N*	0.10	0.20	0.04	0.28	0.07	0.18	0.23	1.00		
MSPSS	−0.14	0.09	0.02	0.15	−0.10	0.34${}^{**}$	0.30${}^{*}$	0.19	1.00	
WEMWBS	0.04	0.19	0.07	0.26	−0.01	0.31${}^{*}$	0.28	0.04	0.39${}^{***}$	1.00

${}^{***}p<.001$, *p*<.01, *p*<.05.
